# Insertion eines zweiten Elektrodenträgers – eine seltene Komplikation bei CI-Reimplantation

**DOI:** 10.1007/s00106-023-01363-1

**Published:** 2023-10-16

**Authors:** M. C. Ketterer, K. Brückerhoff, S. Arndt, R. Beck, A. Aschendorff

**Affiliations:** Klinik für Hals‑, Nasen‑, Ohrenheilkunde, Universitätsklinikum Freiburg, Medizinische Fakultät, Albert-Ludwigs-Universität Freiburg, Killianstraße 5, 79106 Freiburg, Deutschland

**Keywords:** Technisches Upgrade, Reimplantation, Komplikation, Zweiter Elektrodenträger, Skaläre Lage, Technical upgrade, Reimplantion, Complication, Second electrode array, Scalar position

## Abstract

Die Notwendigkeit der Explantation eines Cochleaimplantats ist sowohl bei technischem Defekt als auch aus medizinischer Indikation möglich. Dieser Fall zeigt, dass bei Reimplantation der Cochlea das Risiko eines nicht luxierbaren Elektrodenträgers, wie hier beschrieben aus der Scala tympani, besteht. Die Insertion eines zweiten Elektrodenträgers in die freie und reizlose Scala vestibuli ist in diesem Fall gelungen. Nichtsdestotrotz muss die Indikation zur Reimplantation insbesondere bei tolerablen Einschränkungen mit nur wenig oder keinem Verlust im Sprachverstehen kritisch gestellt werden und sollte nicht allein aufgrund eines gewünschten Implantat-Upgrades durchgeführt werden.

## Abstract (en)

Due to a technical defect or a medical indication, it may be necessary to explant a cochlear implant. This case report shows that there is the risk of encountering a nonremovable electrode array—as described here from the scala tympani—during cochlear reimplantation. In the present case, insertion of a second electrode array into the free and nonobstructed scala vestibuli was successful. Nonetheless, the indication for reimplantation must be carefully considered, especially in patients with tolerable limitations with little or no loss of speech understanding. Furthermore, surgery should not be performed solely because an implant upgrade is desired.

## Anamnese

Eine 22-jährige Patientin stellte sich 20 Jahre nach Erstimplantation eines Cochleaimplantats (CI; 22 + 10 Cochlear^TM^, Cochlear Limited, NSW, Sidney, Australien) auf der linken Seite mit zunehmenden Schmerzen im Bereich des Implantatlagers, insbesondere bei Tragen des Sprachprozessors, vor. Der Höreindruck sei gleichbleibend, jedoch werden subjektiv Störgeräusche beim Tragen des Prozessors beschrieben, sodass die Patientin diesen seltener trage. Aufgrund einer Implantatdislokation nach anterior Richtung Mastoidrand führten wir bereits zehn Jahre zuvor eine Revisionoperation mit Dorsal- und Kaudalverlagerung durch. Die Patientin leidet an einer beidseitigen kongenitalen Innenohrschwerhörigkeit unbekannter Genese. Eine syndromale Schwerhörigkeit wurde ausgeschlossen; Schwangerschaft und Geburt verliefen ohne Komplikationen. Eine familiäre Schwerhörigkeit liegt nicht vor. Rezidivierende Otitiden, Otorrhö sowie Vertigo und Tinnitus werden verneint.

## Befund

Bei Aufnahme zeigen sich Gehörgänge und Trommelfell beidseits reizlos. Das Implantatlager war linksseitig kaudaler als üblich tastbar und ohne Anhalt auf Infektion oder Hämatom. Im Freiburger Sprachtest ergaben sich ein Einsilberverstehen von 50 % bei 65 dB und ein Zahlenverstehen von 90 % mit CI.

## Diagnose

Bei Verdacht auf „soft failure“ sowie subjektiv wahrgenommene Störgeräusche bei unverändertem Sprachverstehen wurde die Indikation zur Revisionsoperation mit Implantatwechsel gestellt.

## Therapie und Verlauf

**Figure 1:**
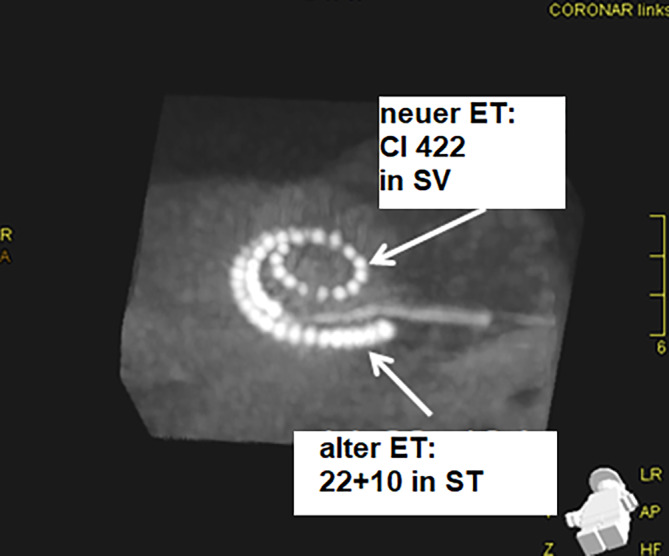

Intraoperativ zeigte sich der Elektrodenträger, welcher ursprünglich über eine Cochleostomie in die Scala tympani inseriert war, nicht mobilisierbar. Grund hierfür war eine reaktive Osteoneogenese, die sich vollständig um den Elektrodenträger ausgebildet hatte, sodass dieser, auch nach Erweiterung der Cochleostomie, nicht entfernt werden konnte. Initial war die Insertion in Soft-Surgery-Technik nach Lehnhardt durchgeführt worden. Es erfolgte die Eröffnung der Scala vestibuli, die sich reizlos und vollständig offen zeigte. Die Insertion einer Probeelektrode gelang vollständig. Die Insertion des geraden 422-Elektrodenträgers (422 Cochlear^TM^) in die Scala vestibuli gelang problemlos, sodass entschieden wurde, den nicht luxierbaren Elektrodenträger in der Scala tympani zu belassen. Dieser wurde gekürzt und die Cochleostomie mit Bindegewebe abgedeckt. Impedanzprüfung, Stapediusreflexschwellen(SRT)-Messung sowie die „neural response telemetry“ (NRT) ergaben regelrechte Werte. Die postoperative Bildgebung mittels Rotationstomographie zeigte die beiden einliegenden Elektrodenträger in Scala vestibuli und Scala tympani (Abb. 1). Das Sprachverstehen nach Reimplantation zeigte sich postoperativ mit 80 % Einsilberverstehen und 100 % Zahlenverständnis und blieb über 4 Jahre nach Reimplantation stabil.

## Diskussion

Die Explantation eines Cochleaimplantats kann bei technischem Defekt des Implantats sowie aus medizinischer Indikation, beispielsweise einer Infektion oder Elektrodenträgerdislokation, notwendig werden. Ein technischer Defekt kann in „hard failure“ und „soft failure“ unterteilt werden. Ein „hard failure“ bezeichnet den Funktionsverlust des Implantats mit objektivierbarem Ausfall in der Integritätstestung. „Soft failure“ beschreibt dagegen ein technisch funktionsfähiges Implantat, welches für den Patienten jedoch keinen ausreichenden Benefit erbringt oder anderweitige Beschwerden verursacht, wie zum Beispiel Schmerzen oder Schwindel [12]. Die Reimplantation eines Cochleaimplantats ist ein operativer Vorgang, der 1985 erstmals als mögliche und erfolgreiche Therapieoption bei Funktionsausfall eines Implantats beschrieben wurde [5]. Die Reimplantationsrate bei „hard“ und „soft failure“ reichen in der bisher veröffentlichten Literatur von 0,5 bis 14,7 %, wobei das Auftreten dieser in der Literatur beschriebenen Komplikation aufgrund der häufigeren Stoßverletzungen des Kopfes bei Kindern höher ist als bei Erwachsenen [12]. Obwohl die Erfolgsrate einer Reimplantation, gemessen am Sprachverstehen nach Revisionsoperation, insgesamt gut ist, erzielen besonders jene Kinder eine bessere Hörleistung mit dem neuen Implantat, welche sich in der sensiblen Phase der Sprachentwicklung befinden [10]. Demgegenüber zeigte eine Arbeit unserer Arbeitsgruppe stabile Ergebnisse nach Reimplantation, jedoch ohne signifikante Verbesserung trotz technischen Upgrades [3]. Zahlreiche Arbeiten zeigten, dass die primäre Insertion des Elektrodenträgers in die Scala tympani zu favorisieren ist und zu signifikant besserem Sprachverstehen führt [2, 4]. Die Insertionsqualität ist von Faktoren wie der cochleären Morphologie [7, 8; 9] und der chirurgischen Lernkurve abhängig [1]. Eine Implantation des Elektrodenträgers in die Scala vestibuli ist dennoch möglich und beispielsweise bei Otosklerose oder Obliteration der Scala tympani auch notwendig. Dies kann dennoch zu einer zufriedenstellenden Hörrehabilitation trotz partieller Ossifikation und Scala-vestibuli-Insertion führen [11]. Die notwendige Scala-vestibuli-Insertion ist jedoch bisher nicht als Folge einer zuvor erfolgten Implantation in die Scala tympani beschrieben worden, sondern als gegebene Voraussetzung vor Elektrodeninsertion, beispielsweise aufgrund Obliteration infolge einer Otosklerose oder Infektion. Die im vorliegenden Fall beschriebene reaktive Verknöcherung der Scala tympani nach Elektrodenträgerinsertion kann durch das operative Trauma an der Hörschnecke, welches eine Entzündungsreaktion mit konsekutiver Osteoneogenese verursacht [6], begründet sein. Darüber hinaus besteht die Möglichkeit einer metachronen, stummen Labyrinthitis mit Ossifikation als Folge. Dennoch ist die Entfernung und Reimplantation eines Elektrodenträgers in der Folge meist komplikationslos möglich [12]. Im vorliegenden Fall trat eine Verknöcherung der Scala tympani mit Unmöglichkeit der Explantation eines Elektrodenträgers auf, die erst intraoperativ zu beobachten war. Ursächlich war neben der Neoossifikation auch die Form des Elektrodenträgers (CI22 + 10, Cochlear^TM^, Elektrodenträger mit Ringelektroden), die eine Luxation unmöglich machte.

Auch bei der heute favorisierten Rundfensterinsertion mit hohem Anteil eines Erhalts von Restgehör und sog. atraumatischem Vorgehen, besteht im Verlauf das Risiko der Obliteration und Ossifikation durch eine stumme Labyrinthitis, wodurch es im Fall der Reimplantation zu unvorhergesehenen Komplikationen kommen kann.

Die Methode der Implantation in die Scala vestibuli ist bekannt, jedoch ist dies der erste Fallbericht über ein solches Vorgehen bei bereits einliegendem und nicht luxierbarem Elektrodenträger in der Scala tympani. Die Patientin erzielte mit dem neuen Implantat eine gute Hörleistung, welche mit dem Sprachverstehen vor Reimplantation vergleichbar ist. Postoperativ ergab sich ein Einsilberverstehen im Freiburger Sprachtest von 80 % bei 65 dB SPL in der Messung ein Jahr nach Reimplantation, welches über 4 Jahre nach Reimplantation stabil blieb.

Dieser Fall zeigt, dass bei Reimplantation der Cochlea das Risiko eines nicht luxierbaren Elektrodenträgers besteht. Die Insertion eines zweiten Elektrodenträgers in eine reizlose Scala vestibuli ist zwar in diesem Fall gelungen, dennoch sollte die Indikation zur Reimplantation kritisch gestellt werden. Eine Reimplantation bei tolerablen Einschränkungen mit nur wenig oder keinem Verlust im Sprachverstehen ist zwar immer eine individuelle Entscheidung, dennoch sollte die Reimplantation nicht allein aufgrund eines gewünschten Implantat-Upgrades durchgeführt werden.
